# Tipping point arises earlier under a multiple-stressor scenario

**DOI:** 10.1038/s41598-023-44012-x

**Published:** 2023-10-05

**Authors:** Charlotte Carrier-Belleau, Ludovic Pascal, Scott D. Tiegs, Christian Nozais, Philippe Archambault

**Affiliations:** 1 Département de biologie, Université Laval, 1045, av. de la Médecine, Quebec, QC G1V 0A6 Canada; 2 Québec-Océan, Université Laval, 1045, av. de la Médecine, Quebec, G1V 0A6 Canada; 3 Takuvik Joint UL/CNRS Laboratory, Université Laval, 1045 Avenue de la Médecine, Quebec, QC G1V 0A6 Canada; 4 Institut des sciences de la mer de Rimouski, Canada Research Chair in geochemistry of coastal hydrogeosystems, Université du Québec à Rimouski, 310 Allée des Ursulines, Rimouski, QC G5L 3A1 Canada; 5 Department of Biological Sciences, Oakland University, Rochester, MI USA; 6 Département de biologie, chimie et géographie, Université du Québec à Rimouski, 300 Allée des Ursulines, Rimouski, QC G5L 3A1 Canada

**Keywords:** Ecology, Environmental sciences, Limnology, Ocean sciences

## Abstract

Anthropogenic impacts and global changes have profound implications for natural ecosystems and may lead to their modification, degradation or collapse. Increases in the intensity of single stressors may create abrupt shifts in biotic responses (i.e. thresholds). The effects of multiple interacting stressors may create non-additive responses, known as synergistic or antagonistic interactions. Here we combine both concepts—ecological thresholds and interactions between multiple stressors—to understand the effects of multiple interacting stressors along environmental gradients, and how this can affect the occurrence of thresholds. Using an experimental approach to investigate the effect of nutrient enrichment and saltwater intrusion on mortality in the zebra mussel, *Dreissena polymorpha*, we show that multiple stressors can create thresholds at lower levels of an environmental gradient. Our results reveal a major shortcoming in how we currently investigate these two ecological concepts, as considering them separately may be causing underestimation of thresholds and stressor-interaction impacts.

## Introduction

Anthropogenic activities substantially modify ecological processes and degrade aquatic ecosystems^1–3^. The origin, magnitude, and cumulative impacts of human activities and global changes threaten environmental systems and represent major challenges for their conservation and management. Often, ecosystems do not respond to gradual changes smoothly and linearly. Instead, increasing evidence suggests that ecosystems under pressure may reach tipping points or thresholds that translate into abrupt shifts from one state to another^4,5^. In addition, the cumulative effects of multiple interacting stressors may lead to “ecological surprises” such as synergistic or antagonistic effects^6–8^.

Threshold effects and interactions between stressors have each been extensively described separately in various ecosystems and found to be significant sources of uncertainty when predicting ecological changes^9,10^. However, studies integrating the two concepts are rare^10^, even though multiple stressors are known to cause abrupt changes in ecosystems when acting in combination. For example, combining pulse “triggers” (e.g. strong storms) to slower change “press” stressors (e.g. gradual warming) may cause a system to switch back and forth between alternative states and eventually cause abrupt and profound changes to the stable state^11–13^. The integration of these two concepts is fundamental to understand both concepts better individually and because their combined effects could have major implications for understanding human impacts on the natural world. However, the combination of both concepts requires significant logistics as well as complex experimental designs.

We therefore need to reconsider our approach to studying stressor interactions and threshold effects because investigations typically evaluate (1) stressor interactions when considering only one level of intensity for each stressor, and (2) thresholds along singular environmental gradients (i.e., without considering multiple stressors). As a proof of concept and to show the importance of combining both concepts, we conducted a laboratory experiment and assessed the potential for stressors to interact along an environmental gradient and test whether pulse stressors may outpace an existing threshold along a single-stressor gradient. We accomplished this by determining the mortality levels of the invasive freshwater zebra mussel *Dreissena polymorpha* (Pallas, 1771) when exposed to a press stressor (nutrient enrichment gradient) for two months in the absence and presence of a pulse stressor (saltwater intrusion). Nutrient (N, P, K) enrichment and saltwater intrusion were chosen because they are common environmental stressors in estuarine coastal and freshwater habitats across the planet, and lead to quantifiable changes in aquatic communities and ecosystem functioning^14^. Our results show that, within a multi-stressor context, earlier (i.e., lower threshold value) and multiple thresholds can emerge along environmental gradients. This finding suggests that we may have underestimated the frequency of threshold dynamics when investigating stressors in isolation, especially in the current context of highly cumulative human impacts in aquatic ecosystems^3^.

## Results and discussion

### Evidence for threshold along gradient

**Figure 1:**
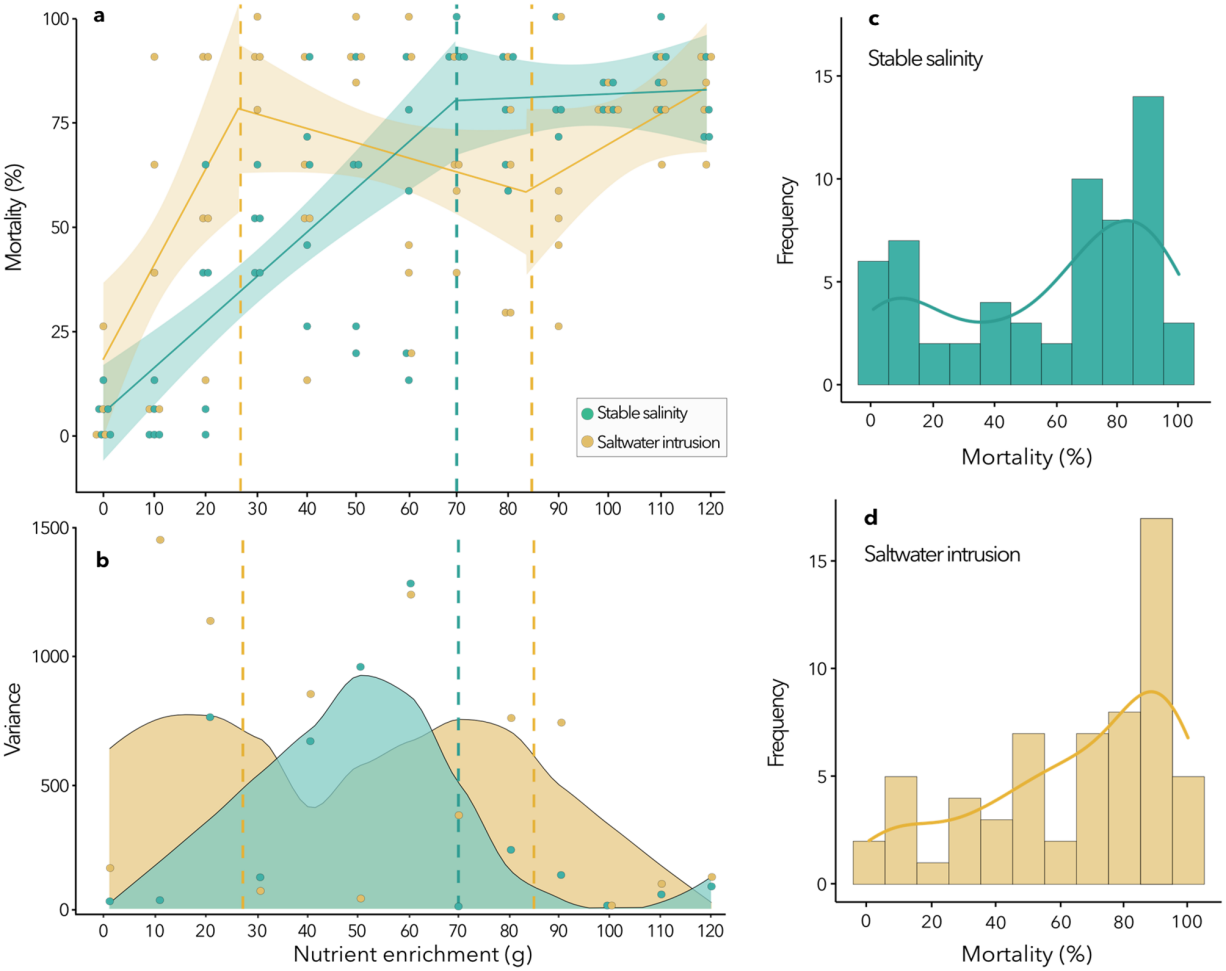
Evidence for an ecological threshold in the context of single (stable salinity in green) and multiple stressors (saltwater intrusion in yellow) for mortality levels in zebra mussel *Dreissena polymorpha* populations along a nutrient enrichment gradient. (**a**) The effect of two months of exposure to a press nutrient enrichment, both alone and combined with saltwater intrusion on the mortality of adult mussels. Points represent raw data for adult mortality observed in individual tanks. Full lines represent broken-line regression models for the stable salinity and saltwater intrusion, and shaded zones represent 95% confidence intervals. Dashed vertical lines represent significant (*p* < 0.05) thresholds identified for the stable salinity and saltwater intrusion. (**b**) Variance points and smooth curve of variance of mortality for each group of observations along the nutrient enrichment gradient for both stable salinity and saltwater intrusion. (**c**,**d**) Frequency distribution of observed mortality in the experimental study (histogram) and density of values along the gradient (curve), where the surface under the curve corresponds to one, in the absence (**c**) and presence (**d**) of saltwater intrusion.

Threshold effects are present for both salinity treatments (i.e., stable salinity, saltwater intrusion) along the nutrient enrichment gradient (Fig. 1a, Supplementary Tables S1, S2). Figure 1a shows mortality in *D. polymorpha* along the tested nutrient enrichment gradient, both in isolation and in combination with a weekly saltwater intrusion, after two months of exposure. Using broken-line linear regression models (SEGMENTED R package)^15,16^, we identify significant thresholds (Davies Test, *p* ≤ 0.05) along our gradient in the presence and absence of saltwater intrusion. The first significant threshold is present at 70.00 g of slow-dissolving fertilizer, used to recreate the nutrient enrichment gradient, without saltwater intrusion (Davies test, *p* = 0.003) (Supplementary Table S3 for corresponding nutrient concentrations). Before this point, mean mortality is always lower at any given fertilizer concentration. However, beyond this threshold, mean mortality increases without saltwater intrusion. Two significant breakpoints for the saltwater intrusion treatment (Davies test, *p* = 0.00378) were identified: one at 26.91 g of fertilizer and a second at 84.13 g. These two thresholds are characterized by an increase in mean mortality from 0 to 30 g, followed by an overall decrease between 30 and 90 g, and finally an increase from 90 to 120 g of fertilizer. The decrease in mean mortality between the two breakpoints in the presence of saltwater intrusion suggests that exposure to nutrient enrichment is optimal between those two concentrations under osmotic stress. Nutrients between 30 and 90 g may have led to increased energy intake through bottom-up increases in phytoplankton and organic matter that benefited mussels exposed to osmotic stress^17^.

These threshold features along the gradient indicate the presence of an ecological tipping point for both salinity treatments. Indeed, strong evidence for tipping points is provided by an increase in variance for a given response when approaching a threshold value^18,19^, as is observed here in the absence of saltwater intrusion (Fig. 1b). This confirms that variance increase could be used as early warning signals for ecosystem management and conservation to identify tipping points along gradients before they arise^19^. The existence of a bimodal or multimodal distribution suggests alternative stable states characterized by the occurrence of a transition ^20^. To assess the multimodality of the magnitudes of the response (i.e., mortality), we used Hartigan’s dip test method^21^. In the presence and absence of saltwater intrusion, we show multimodality in the frequency distribution of the observed mortality (*p* = 0.039 for both salinity treatments, Fig. 1c,d). Therefore, increasing variance before the identified thresholds and a multimodal frequency distribution of observed mortality are compelling evidence of tipping points. Moreover, these results indicate that the overlap of multiple stressors in a system decreases threshold values along the gradient and induces multiple tipping points. This novel finding questions the usefulness of identifying tipping points along singular gradients, given the current cumulative exposure in aquatic ecosystems^3^, when attempting to provide specific thresholds for conserving and managing aquatic ecosystems and resources.

### Interactions of stressors along the nutrient enrichment gradient

**Figure 2:**
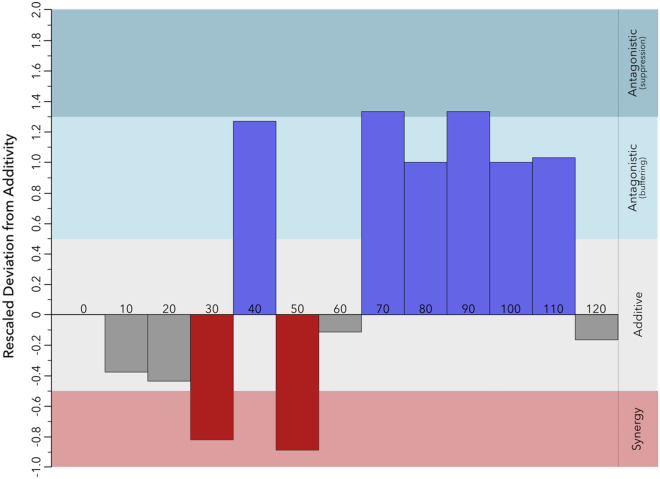
Stressor interactions along a nutrient enrichment gradient for mean mortality in the zebra mussel *Dreissena polymorpha* after two months. The Rescaled Deviation from Additivity metric shows the interaction types and magnitude between saltwater intrusion and nutrient enrichment at different concentrations. Grey bars represent additive responses, red bars represent synergies (greater than the null-hypothesis of no-interaction) and blue bars represent antagonistic interactions (weaker than the null-hypothesis of no-interaction). Values range from − 1 to 2 where − 1 indicates an extremely synergistic interaction and 2 an extremely antagonistic interaction. Values ranging from − 0.5 to 0.5 indicate additive responses.

**Figure 3:**
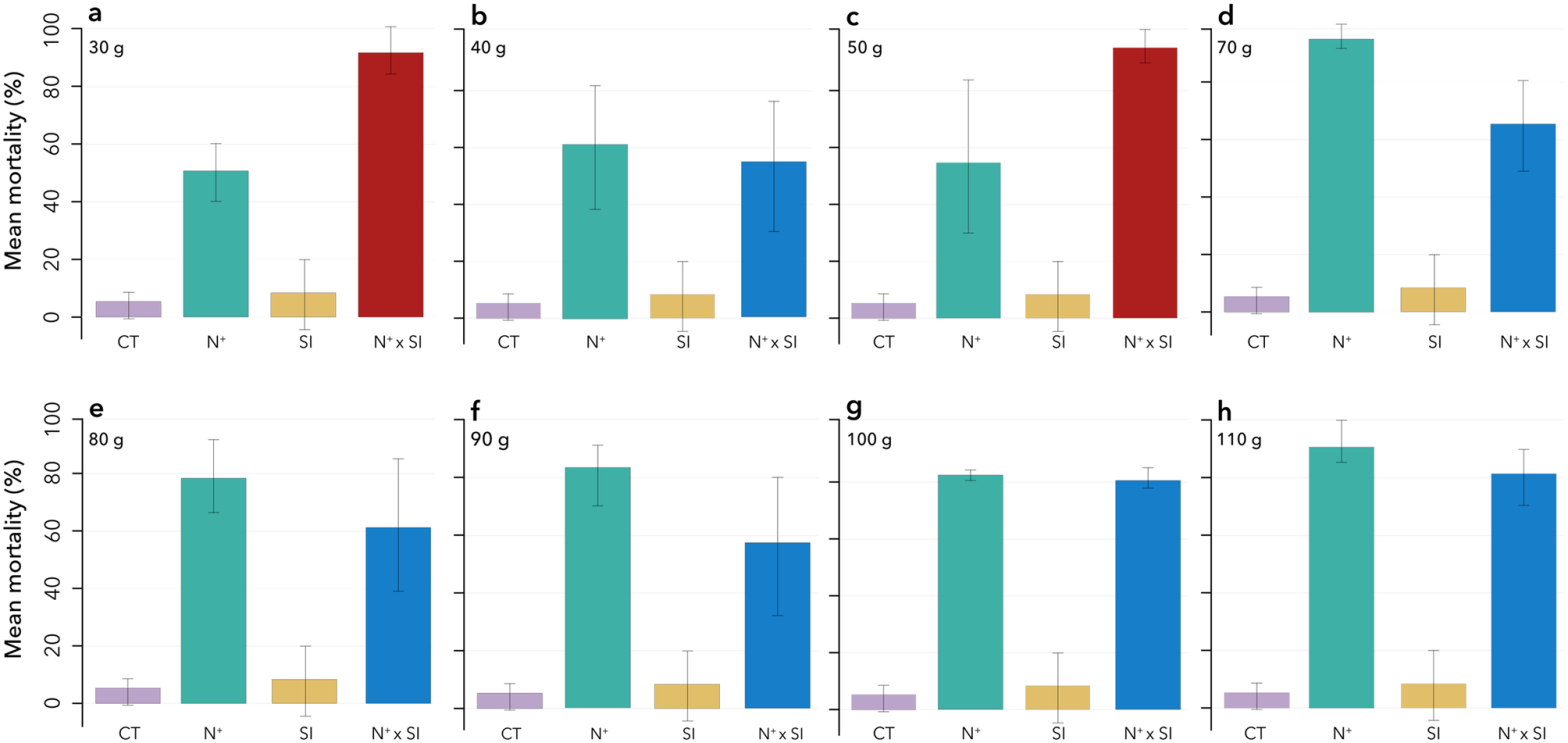
Mean mortality associated with the different treatments along the nutrient enrichment gradient: control (CT), nutrient enrichment at a given concentration (N^+^), saltwater intrusion (SI) and combined effect of nutrient enrichment and saltwater intrusion (N^+^ × SI). Error bars correspond to the 95% confidence interval. Results are shown for the nutrient concentrations where interactions (i.e., synergies or antagonisms) occurred between the two stressors.

Mortality levels in *D. polymorpha* due to nutrient enrichment are modified by the presence of saltwater intrusion, as indicated by the presence of a significant interaction between those two factors (*F*_12,91_ = 7.799, *p*-value < 0.0001) (Supplementary Table S4). This suggests that stressors may interact either synergistically and/or antagonistically along the gradient. The Rescaled Deviation from Additivity^22^ allows us to visualize the interaction types and to interpret the magnitude of the interactions along the gradient. These results are shown in Fig. 2, where the top quadrant represents antagonistic interactions (buffering or suppression) and the bottom quadrant represents synergies. Synergies where the combined effect of both stressors is superior to a null model (see Methods), resulting in an accentuated effect (i.e., higher mortality) as well as additive responses are prevalent at lower concentrations of nutrients along the gradient (Figs. 2, 3). However, as we reach the tipping point under stable salinity conditions (70 g), a switch occurs, and interactions become antagonistic (Figs. 2, 3). Indeed, the combined effects of salinity and nutrient enrichment on mussel mortality are less than the null model and result in an attenuated effect (i.e., lower mortality). Consequently, under conditions of low nutrient enrichment, saltwater intrusion exacerbates the mortality in *D. polymorpha*, generating a lower threshold value compared to conditions of stable salinity.

The individual effects of elevated nutrient concentrations and saltwater intrusion on bivalves are well-known, allowing us to interpret their combined effect, especially synergistic interactions. First, increased nutrient concentrations may directly or indirectly affect marine bivalves. An indirect effect arises through the proliferation of primary producers (e.g. phytoplankton, microphytobenthos) that will ultimately increase fluxes of organic carbon and increase organic matter decomposition^23,24^. In certain circumstances, this will increase oxygen demand near sediments through respiratory processes and lead to low dissolved oxygen concentrations^23,24^ (see Supplementary Table S3). Whilst organisms may show tolerance and resistance, they will ultimately alter fundamental behavioral or physiological patterns and increased mortality levels may be observed^22^. Nutrient input can also have direct physiological impacts on aquatic organisms. For instance, high nutrient concentrations can alter the acid–base and ionic balance in freshwater bivalves which results in high energetic costs for organisms^23^. This is mainly explained by the toxicity of inorganic nitrogenous compounds^23^ and result in subsequent physiological consequences such as lower metabolic rate, tissue growth, and suppressed immune responses^25–27^. Concerning saltwater input, studies have indicated that *D. polymorpha* is a poor hyperosmotic regulator compared to other freshwater bivalves^28^. When exposed to an osmotic stress, salinities may be similar to those in tissues and there may be an osmotic water loss and an active uptake of ions from the solution^29,30^. This energy invested in tissue proteins use and shell material to maintain osmotic balance may result in less energy available to maintain other fundamental physiological processes^31–33^. Salinity variation has also been shown to alter metabolic rates^34^, that can ultimately translate in higher mortality levels. The combined effect of the environmental stressors may affect different physiological pathways and have varying effects on responses measured at the individual level, as seen in different species of marine bivalves^17^. The antagonistic relationships are mainly explained by the fact that at these nutrient concentrations, the effect of one stressor (i.e., nutrient enrichment) is entirely masking the effect of another stressor and both stressors combined. More specifically, at specific nutrient concentrations (i.e., 70 and 90 g), extreme forms of antagonisms, also known as suppressions, have been identified^22^. In this case, nutrient enrichment does not mask the effect of saltwater intrusion but actually reverses the adverse effects of saltwater intrusion.

## Conclusion

We have shown that multiple stressors (i.e. nutrient enrichment and saltwater intrusion) can interact differently as they progress along an environmental gradient, acting, in this case, synergistically under relatively low nutrient pressure and antagonistically at increasing nutrient concentrations. Synergistic interactions, and the enhanced effect this interaction creates, can generate earlier tipping points in ecological systems. Our results have two main implications regarding our ability to provide conservation and management action guidelines. First, knowing the type and intensity of interactions among stressors may help maximize environmental management outcomes^8^, particularly as varying interactions along gradients greatly complexifies the work of conservationists and environmental managers. Ecosystem spatial and temporal gradients may derive from human activities or global natural phenomena. A local stressor may, thus, interact differently depending on the intensity of another local or global stressor, making it highly challenging for practitioners to predict and manage the combined effect of multiple stressors. Second, our results showed that adding a second stressor in an ecosystem may result in an outpaced ecological threshold. A recent meta-analysis has shown that thresholds for ecological responses to global change do not emerge from empirical data^20^. However, this meta-analysis considered unique environmental gradients and questions the relevance of considering simple scenarios and single gradients to identify thresholds to guide management actions^13^, especially since most aquatic habitats are now exposed to multiple stressors (e.g. pulses and presses of stressors)^3^. Considering both concepts—stressor interactions and thresholds—may provide a holistic view of ecosystem sensitivity to environmental stressors and help prevent the loss of ecosystem services and functions.

## Material and methods

### Specimen collection

We investigated the mortality in *D. polymorpha*, as its sedentary behaviour makes it particularly susceptible to an increasing number of environmental stressors. We deployed 36 sets of 14 7.62 cm round Masonite disks (Hester Dendy, NKY environmental supply, Kentucky, USA) in the Bassin Louise (46° 31′ N, 71° 20′ W) in Quebec City (QC, CA) from June to October 2020 to allow colonization of *D. polymorpha*. Plates were then retrieved and transferred to Université Laval where 3,500 individuals (less than 9 mm) were transferred to ten 37 L aquaria. These served as temporary aquaria for one-month acclimation to laboratory conditions (8 °C). Each aquarium was equipped with an Air Pump (Marina 50, 11110, Hagen) and a filter (Marina Slim Filter S10, A285) and was exposed to a 12 h photoperiod (7:00 AM–7:00 PM). Organisms were fed daily with Shellfish Diet 1800 (Reed mariculture, Campbell, CA, USA) – a mix of six marine microalgae: *Isochrysis*, *Pavlova*, *Tetraselmis*, *Chaeotoceros calcitrans*, *Thalassiosira weissflogii* and *Thalassiosira pseudonana*^32^.

### Experimental treatments, design, and system

We performed a two-month experiment from November 16th, 2020 to January 11th, 2021. We tested the effect of nutrient enrichment, and nutrient enrichment coupled with salinity variation, on mortality levels in *D. polymorpha* by randomly assigning each experimental unit (3.79 L plastic aquaria) to one of the 26 experimental treatments following a full-factorial design (*n* = 5) (Supplementary Table S2)^32^. Fifteen individuals of *D. polymorpha* were placed in every aquarium. The flow-through experimental set-up was composed of 130 aquaria randomly distributed in 6 water baths to maintain a constant temperature of 8 °C. The aquaria and water baths were supplied by the freshwater reserve at LARSA that is supplied by a municipal water intake. The water first passed through a sand filter (MAR-24-0, Culligan, QC, Canada) to eliminate all suspended particles, then continued to a dechlorinator (MAR-C-42, Culligan, QC, Canada). Finally, a UV filter acted as a third filtration system to kill any pathogens that resisted the previous treatments.

To recreate a 13-level nutrient enrichment gradient, we used an in situ enrichment technique with controlled-release pellets in mesh bags (Acer NT NPK 17-7-10, Plant-Prod, Brampton, ON, Canada)^17,32,33^. A eutrophication gradient was recreated by using 13 concentrations of phosphorus and nitrogen (Supplementary Table S3). The fertilizing pellets were placed in mesh bags attached from the surface of the aquarium. Nutrient percentage loss did not differ across treatments (Supplementary Table S5) and was similar to other similar experiments that simulated nutrient enrichment^17,32,34^. The nutrient concentrations were based on realistic conditions measured in Quebec, and mimicked a freshwater ecosystem affected by agricultural nutrient enrichment, which changes from a mesotrophic to a eutrophic state^35^. For example, mean phosphorus and nitrogen concentration in the region of Quebec in 2019 were respectively 1.10 and 43.57 µmol/L with anomalies reaching 2.84 µmol/L and 80.68 µmol/L^36^. Nitrate and phosphate concentrations were measured twice during the experiment (December 14th, January 8th) using a modified protocol from Grasshoff and colleagues^37^. To control for the potential effect of the mesh bags in the aquaria, we place mesh bags filled with aquarium pebbles in our control treatments (no nutrient enrichment).

To examine how a second stressor could alter the effect of a tipping point in a freshwater ecosystem, half of the aquariums were also exposed to saltwater input. We induced an osmotic stress once per week by adding 400 mL of artificial saltwater (i.e., with mineral chemical and inorganic compounds) to attain 30 PSU. A mean salinity of 3 PSU (Supplementary Table S2) for four hours was obtained in those experimental units. This treatment represented the shifting of a salinity front, as is seen in natural estuaries^38^. For control conditions, we placed mesh bags filled with aquarium gravel and 400 mL of freshwater in the aquaria with stable salinity, to control for the possible effect of increased flow^32^.

An extra set of aquaria containing 100 individuals of *D. polymorpha* for every treatment was used to replace dead individuals in the experimental aquaria to keep a constant density of individuals. Temperature, pH, and salinity were measured daily (Supplementary Table S3).

### Mortality levels

Mortality is defined as the percentage of dead individuals compared to those initially present. It was counted in every aquarium at the end of the experiment by identifying the total number of dead, tagged individuals throughout the experiment. Once a week, we searched for dead individuals to be retrieved from the aquaria and replaced by live individuals of *D. polymorpha* that were taken from aquaria under the same experimental conditions. However, mortality only occurred during the last week of the experiment. Adjusted means (considering the effect of the water baths) and means for mortality levels of *D. polymorpha* for all treatments are shown in Supplementary Tables S4 and S5.

### Breakpoint analysis and evidence for tipping point

We used broken-line regression models to find the breakpoints for mortality levels in *D. polymorpha* (SEGMENTED R package)^15,16^. This analysis determined the relationship between mortality levels and nutrient enrichment for both salinity treatments and allows us to observe changes in the regression relationship. We then estimated the breakpoint by assessing a relevant gap and a difference in slope coefficient in the linear predictor^16^. To test if the slopes before and after the breakpoint were significantly different (*p* ≤ 0.05), we used the Davies Test^39^. If the p-value of the Davies Test < 0.05, this demonstrates that the breakpoints regressions are significantly different from a single linear regression of al points. Breakpoint analysis is highly susceptible to data extremes. We thus identified and excluded outliers from the analysis using Dixon’s test^40^. To strengthen the evidence of a tipping point, we assessed the variability of mortality rates for every nutrient concentration along the gradient for both salinity treatments. We then used a smoothing curve to describe the relationship between mortality variance and nutrients for both salinity treatments. If the data were organized around a tipping point, it would be expected that variance would increase in response variables as the pressure strength reaches or crosses the threshold value^18,19^. Finally, we used Hartingan’s dip test method (HD) to assess the multimodality of effect sizes^21^. If significant, the HD statistical test indicates that a frequency distribution has more than one mode, indicating the presence of two (or more) alternative ecosystem states.

### Effect of stressors and interaction classification

To characterize the interaction type, distinguishing between additive responses, synergies and antagonism we used the Rescaled Bliss Independence Model (RBI)^22^. This newly introduced framework has been proposed to replace ANOVA, which has limitations regarding the detection of interactions^22^. The data was first converted to survival percentages (100—mortality percentage). We then calculated the relative response (w_x_) in our simplified assemblages by dividing the absolute stressor response measurements (W_x_) by the response observed in control conditions (W_0_—no nutrient enrichment, no saltwater intrusion). We then characterized the effect of a single (X or Y) or combined stressor (XY) by comparing it to control conditions and denoted this relative response in the presence of a stressor (X or Y) by w_X_ or w_Y_ or w_XY_. The unscaled interaction metric (also referred to Deviation from Additivity—DA) was then calculated using the following formula:

$${DA =w}_{XY}-{w}_{X}{w}_{Y}$$

We then applied the rescaling method described in Tekin et al. 2020 to calculate interaction values and to determine the interaction type (Rescaled DA). This led to distinct values ranging from − 1 to 2. Values ranging from − 1 to − 0.5 were deemed to be synergistic interactions, from − 0.5 to 0.5 to be additive responses, and from 0.5 to 2 antagonistic interactions. More specifically, values ranging from 0.5 to 1.3 were deemed to be antagonistic buffering interactions, where the combined effect of multiple stressor does not differ much from the strongest single stressor and values ranging from 1.3 to 2 were categorized as an extreme form of an antagonism, also called a suppression. In this case, one stressor reverses the effect of another stressor.

As ANOVAs have historically been used to determine stressor interactions, the single and combined effects of nutrient enrichment and salinity variation were also analyzed using a two-way heterogeneous variance mixed model with nutrient enrichment (13 levels) and salinity variation (2 levels) as fixed factors, and a water bath as a random factor^40^. This was done to ensure continuity between the two statistical approaches. To test for the effect of the random factor, we performed a variance component test with the log-likelihoods of the model with and without the random factor (i.e., water bath) (varCompTest function of the varTestnlme package in R). The water bath had no significant effect (*p* < 0.1). We performed a Shapiro–Wilk test to verify the assumption of normal distribution and used regression diagnostic plots to verify the homoscedasticity of residuals (equal variance of residuals). We set the possibility of making a Type I error to α = 0.05. The adjusted means for all treatments are displayed in Table S4.1. We then used pair-wise comparisons between nutrient enrichment and salinity treatments to understand which levels differed. We then identified (1) the mean mortality in our control (2) the effect of nutrient enrichment on mortality (i.e. in comparison to the control) and (3) the effect of salinity variation on mortality and (4) the observed combined effect of both salinity variation and nutrient enrichment.

To interpret the nature of the interactions, we compared our observed combined effect of both stressors at a given nutrient concentration and compared that combined effect to a multiplicative effects model^41^. We characterized the interaction type (synergism and antagonism) and the response magnitude and direction (positive/negative synergism or antagonism) of stressors’ combined effect relative to the effect observed under control conditions (no nutrient, stable salinity). A multiplicative null model was used to correct the situation that individuals killed by a factor cannot be killed by another and set the mortality to a maximum of 100%^8^.

$$Multiplicative\, null \,model=\left(\left(N+SV\right)-\left(N \times SV\right)\right)$$

where N and SV correspond to the effects of nutrient enrichment at a given concentration and salinity variation, respectively. These results are similar to the ones obtained using the Rescaled Bliss Independence Model and are shown in Supplementary Table S4 and Figure S1.

### Supplementary Information

Supplementary Information.

## Data Availability

The datasets used and analyzed during the current study is available from the corresponding author on reasonable request. The data will also be available online after publication.
