# Supplementary material for: Tipping point arises earlier under a multiple-stressor scenario
Source: Sci Rep. 2023 Oct 5;13:16780. doi: 10.1038/s41598-023-44012-x (PMC10555998; doi:10.1038/s41598-023-44012-x)
Supplement: Supplementary file 1 — Supplementary Information. [file 41598_2023_44012_MOESM1_ESM.docx]

**Supplementary material for the manuscript entitled «Tipping point arises earlier under a multiple-stressor scenario»**

**Supplementary Table S1:** Estimated break-point(s) and standard error along nutrient enrichment gradient in the absence and presence of saltwater intrusion.

|  | **Est.** | **Std. Err** |
| --- | --- | --- |
| **Stable salinity** |  |  |
| Point 1 | 70.0005 | 9.713 |
| **Saltwater intrusion** |  |  |
| Point 1 | 26.908 | 5.789 |
| Point 2 | 84.130 | 12.164 |
|  |  |  |

**Supplementary Table S2:** Output of the summary.segmented() with information including the meaningful coefficients of the linear terms including the estimate, the standard error (Std. Error), the t value for the “gap” between the fitted lines coming from the model and the p-value associated with the value in the t value column (Pr(>|t|)). Results are also shown for the estimates of the slopes obtained with the slope() function (package “stat”) and the lower (Cl(5%).l) and upper (Cl(5%).u) confidence interval.

|  | **Estimate** | **Std. Error** | **t value** | **Pr(>\|t\|)** | **CI(5%).l** | **CI(5%).u** |
| --- | --- | --- | --- | --- | --- | --- |
| **Stable salinity** | | | | | | |
| Adjusted R-squared = 0.6651 | | | | | | |
| Multiple R-squared = 0.6818 | | | | | | |
| (Intercept) | 5.2940 | 6.0139 | 0.880 | 0.382 | NA | NA |
| Nutrient | 1.0996 | 0.1668 | 6.592 | 1.52e-08 | NA | NA |
| U1. Nutrient | -1.0464 | 0.2830 | -3.697 | NA | NA | NA |
| Slope 1 | 1.1000 | 0.1669 | 6.5925 | NA | 0.76558 | 1.43360 |
| Slope 2 | 0.0532 | 0.2287 | 0.2327 | NA | -0.40466 | 0.51106 |
| **Saltwater intrusion** | | | | | | |
| Adjusted R-squared = 0.3318 | | | | | | |
| Multiple R-squared = 0.3875 | | | | | | |
| (Intercept) | 11.4667 | 11.0175 | 1.041 | 0.30253 | NA | NA |
| Nutrient | 2.6187 | 0.8245 | 3.176 | 0.00245 | NA | NA |
| U1. Nutrient | -3.0277 | 0.8689 | -3.485 | NA | NA | NA |
| U2. Nutrient | 1.2261 | 0.5662 | 2.165 | NA | NA | NA |
| Slope 1 | 2.6187 | 0.8245 | 3.176 | NA | 0.96639 | 4.27090 |
| Slope 2 | -0.4090 | 0.2743 | -1.491 | NA | -0.95869 | 0.14063 |
| Slope 3 | 0.8170 | 0.4953 | 0.495 | NA | -0.17564 | 1.80970 |

| **Treatments** | | **Nutrient concentrations (µmol/L)** | | **Temperature**  **(°C)** | **Salinity during saltwater input (PSU)** | **pH** | **pH during saltwater input** | **Dissolved oxygen during light exposure (%)** | **Dissolved oxygen before light exposure (%)** |
| --- | --- | --- | --- | --- | --- | --- | --- | --- | --- |
| **Nutrient** | **Salinity** | **Phosphate** | **Nitrate** |  |  |  |  |  |  |
| 0 | Stable | 0.87 (± 0.029) | 24.06 (± 0.208) | 8.34 (± 0.006) | 0.00 (± 0.000) | 6.91 (± 0.014) | - | 94.01 (± 0.205) | 92.00 (± 0.252) |
| 10 | Stable | 1.08 (± 0.035) | 26.27 (± 0.238) | 8.37 (± 0.006) | 0.00 (± 0.000) | 6.91 (± 0.012) | - | 93.97 (± 0.207) | 85.07 (± 0.213) |
| 20 | Stable | 1.15 (± 0.048) | 26.84 (± 0.402) | 8.36 (± 0.005) | 0.00 (± 0.000) | 6.90 (± 0.011) | - | 93.87 (± 0.215) | 80.77 (± 0.210) |
| 30 | Stable | 1.76 (± 0.137) | 32.80 (± 1.058) | 8.37 (± 0.005) | 0.00 (± 0.000) | 6.89 (± 0.011) | - | 94.00 (± 0.190) | 80.00 (± 0.218) |
| 40 | Stable | 2.28 (± 0.089) | 35.83 (± 0.619) | 8.37 (± 0.005) | 0.00 (± 0.000) | 6.89 (± 0.011) | - | 93.83 (± 0.216) | 79.48 (± 0.203) |
| 50 | Stable | 2.41 (± 0.118) | 39.31 (± 1.094) | 8.37 (± 0.005) | 0.00 (± 0.000) | 6.90 (± 0.012) | - | 94.06 (± 0.210) | 72.20 (± 0.167) |
| 60 | Stable | 3.27 (± 0.504) | 45.17 (± 3.538) | 8.37 (± 0.005) | 0.00 (± 0.000) | 6.91 (± 0.011) | - | 94.19 (± 0.199) | 72.31 (± 0.179) |
| 70 | Stable | 4.17 (± 0.719) | 48.47 (± 3.634) | 8.37 (± 0.005) | 0.00 (± 0.000) | 6.90 (± 0.014) | - | 94.15 (± 0.206) | 70.52 (± 0.172) |
| 80 | Stable | 4.05 (± 0.961) | 48.48 (± 5.855) | 8.36 (± 0.006) | 0.00 (± 0.000) | 6.91 (± 0.011) | - | 93.99 (± 0.205) | 69.88 (± 0.206) |
| 90 | Stable | 5.64 (± 1.613) | 61.74 (± 9.366) | 8.36 (± 0.006) | 0.00 (± 0.000) | 6.90 (± 0.012) | - | 94.58 (± 0.212) | 66.58 (± 0.180) |
| 100 | Stable | 6.36 (± 2.014) | 63.69 (± 11.136) | 8.37 (± 0.005) | 0.00 (± 0.000) | 6.88 (± 0.011) | - | 93.90 (± 0.208) | 60.01 (± 0.156) |
| 110 | Stable | 6.27 (± 0.515) | 67.92 (± 4.104) | 8.35 (± 0.006) | 0.00 (± 0.000) | 6.90 (± 0.012) | - | 94.29 (± 0.209) | 61.87 (± 0.127) |
| 120 | Stable | 6.45 (± 0.795) | 69.40 (± 5.678) | 8.37 (± 0.005) | 0.00 (± 0.000) | 6.91 (± 0.012) | - | 94.13 (± 0.209) | 59.20 (± 0.111) |
| 0 | Variable | 0.85 (± 0.024) | 23.95 (± 0.095) | 8.36 (± 0.006) | 3.17 (± 0.021) | 6.90 (± 0.011) | 6.97 (± 0.010) | 94.09 (± 0.207) | 90.69 (± 0.255) |
| 10 | Variable | 1.22 (± 0.101) | 27.28 (± 1.074) | 8.36 (± 0.006) | 3.12 (± 0.014) | 6.89 (± 0.011) | 6.97 (± 0.010) | 94.10 (± 0.204) | 83.95 (± 0.184) |
| 20 | Variable | 1.81 (± 0.214) | 30.00 (± 0.722) | 8.36 (± 0.006) | 3.13 (± 0.015) | 6.91 (± 0.009) | 6.97 (± 0.010) | 93.85 (± 0.215) | 80.83 (± 0.213) |
| 30 | Variable | 2.15 (± 0.396) | 35.31 (± 3.394) | 8.36 (± 0.006) | 3.14 (± 0.013) | 6.89 (± 0.013) | 6.97 (± 0.009) | 94.32 (± 0.207) | 79.17 (± 0.196) |
| 40 | Variable | 2.43 (± 0.290) | 37.90 (± 1.853) | 8.36 (± 0.006) | 3.14 (± 0.012) | 6.90 (± 0.014) | 6.98 (± 0.010) | 94.04 (± 0.211) | 79.84 (± 0.180) |
| 50 | Variable | 2.59 (± 0.413) | 40.70 (± 3.232) | 8.37 (± 0.005) | 3.13 (± 0.015) | 6.91 (± 0.012) | 6.97 (± 0.012) | 94.17 (± 0.214) | 72.26 (± 0.157) |
| 60 | Variable | 2.99 (± 0.457) | 41.24 (± 3.471) | 8.37 (± 0.005) | 3.13 (± 0.014) | 6.90 (± 0.008) | 6.97 (± 0.010) | 93.80 (± 0.200) | 72.75 (± 0.183) |
| 70 | Variable | 3.80 (± 0.440) | 48.75 (± 3.678) | 8.36 (± 0.005) | 3.15 (± 0.011) | 6.92 (± 0.015) | 6.96 (± 0.010) | 93.93 (± 0.207) | 70.39 (± 0.187) |
| 80 | Variable | 3.10 (± 0.279) | 46.72 (± 2.322) | 8.36 (± 0.006) | 3.12 (± 0.014) | 6.93 (± 0.012) | 6.96 (± 0.010) | 94.47 (± 0.210) | 67.29 (± 0.209) |
| 90 | Variable | 3.48 (± 0.332) | 50.88 (± 5.294) | 8.37 (± 0.005) | 3.11 (± 0.210) | 6.90 (± 0.011) | 6.98 (± 0.011) | 94.02 (± 0.209) | 66.52 (± 0.471) |
| 100 | Variable | 5.64 (± 0.520) | 62.63 (± 4.168) | 8.37 (± 0.005) | 3.15 (± 0.010) | 6.89 (± 0.009) | 6.98 (± 0.010) | 94.11 (± 0.192) | 58.29 (± 0.216) |
| 110 | Variable | 6.96 (± 1.606) | 68.74 (± 9.272) | 8.36 (± 0.005) | 3.14 (± 0.011) | 6.91 (± 0.011) | 6.99 (± 0.010) | 94.20 (± 0.215) | 60.49 (± 0.221) |
| 120 | Variable | 7.10 (± 1.564) | 69.35 (± 10.524) | 8.37 (± 0.005) | 3.12 (± 0.016) | 6.92 (± 0.008) | 6.97 (± 0.010) | 94.06 (± 0.216) | 58.91 (± 0.102) |

**Supplementary Table S3:** Mean (± SE) values of experimental parameters measured or calculated in aquaria over the duration of the experiments (57 measurements) for nutrient enrichment treatments (Nutrient, in initial g of fertilizer pellets) and salinity variation (Salinity, stable of variable): phosphate and nitrate concentrations, temperature (°C) in aquariums for stable salinity and during saltwater input, salinity (PSU) during saltwater input, pH in aquariums for stable salinity and during saltwater input, and dissolved oxygen during and before light exposure. (%).

**Supplementary Table S4:** Results of the two-way heterogeneous variance mixed model investigating the effect of nutrient enrichment (13 levels) and salinity variation (2 levels) on mortality in *Dreissena polymorpha*. The factors “Nutrient enrichment” and “Salinity variation” were set as fixed and “Water bath” as random but did not have any significant effect. Details are provided for the number of treatment levels - 1 (df1), degrees of freedom for the error (number of observations – number of groups) (df2) F ratio and *p*-value.

| **Source of variation** | **df1** | **df2** | **F ratio** | ***p*-value** |
| --- | --- | --- | --- | --- |
| Nutrient enrichment | 12 | 91 | 64.276 | < 0.0001 |
| Salinity variation | 1 | 91 | 2.080 | 0.1527 |
| Nutrient x Salinity variation | 12 | 91 | 7.799 | < 0.0001 |

**Supplementary Table S5:** Mean (±SE) of initial and final mass (in g) of fertilizing pellets and percentage loss for all experimental treatments.

| **Nutrient** | **Salinity** | **Initial quantity of fertilizing pellets (g)** | | **Final quantity of fertilizing pellets (g)** | | **Loss (%)** | |
| --- | --- | --- | --- | --- | --- | --- | --- |
|  |  | **Mean** | **SE** | **Mean** | **SE** | **Mean** | **SE** |
| 0 | Stable | – | – | – | – | – | – |
| 10 | Stable | 10.0321 | 0.0103 | 7.9880 | 0.1286 | 20.38 | 1.2517 |
| 20 | Stable | 20.0611 | 0.0134 | 15.8900 | 0.2065 | 20.79 | 1.0157 |
| 30 | Stable | 30.0521 | 0.0135 | 23.9180 | 0.2210 | 20.41 | 0.7137 |
| 40 | Stable | 40.0443 | 0.0126 | 32.5880 | 0.4262 | 18.62 | 1.0550 |
| 50 | Stable | 50.0400 | 0.0156 | 39.6780 | 0.1544 | 20.71 | 0.3071 |
| 60 | Stable | 60.0269 | 0.0087 | 47.2520 | 0.3760 | 21.28 | 0.6236 |
| 70 | Stable | 70.0368 | 0.0116 | 56.2720 | 0.4982 | 19.65 | 0.7027 |
| 80 | Stable | 80.0727 | 0.0057 | 63.7280 | 0.4678 | 20.41 | 0.5864 |
| 90 | Stable | 90.0380 | 0.0123 | 72.0760 | 0.4048 | 19.95 | 0.4447 |
| 100 | Stable | 100.0612 | 0.0138 | 80.3180 | 0.4204 | 19.73 | 0.4134 |
| 110 | Stable | 110.0297 | 0.0122 | 87.2120 | 0.6156 | 20.74 | 0.5512 |
| 120 | Stable | 120.0671 | 0.0123 | 96.1740 | 0.5762 | 19.90 | 0.4844 |
| 0 | Variable | – | – | – | – | – | – |
| 10 | Variable | 10.0606 | 0.0093 | 7.6820 | 0.2550 | 23.64 | 2.5270 |
| 20 | Variable | 20.0424 | 0.0104 | 15.7060 | 0.3611 | 21.64 | 1.8097 |
| 30 | Variable | 30.0431 | 0.0148 | 23.6740 | 0.3423 | 21.20 | 1.1551 |
| 40 | Variable | 40.0478 | 0.0168 | 32.2540 | 0.2594 | 19.46 | 0.6560 |
| 50 | Variable | 50.0299 | 0.0030 | 39.6300 | 0.4792 | 20.79 | 0.9552 |
| 60 | Variable | 60.0246 | 0.0038 | 47.8320 | 0.3628 | 20.31 | 0.6005 |
| 70 | Variable | 70.0498 | 0.0180 | 56.1600 | 0.3258 | 19.83 | 0.4533 |
| 80 | Variable | 80.0369 | 0.0132 | 63.9420 | 0.4356 | 20.11 | 0.5506 |
| 90 | Variable | 90.0696 | 0.0092 | 72.2900 | 0.2651 | 19.74 | 0.3015 |
| 100 | Variable | 100.0197 | 0.0050 | 79.6680 | 0.4808 | 20.35 | 0.4811 |
| 110 | Variable | 110.0500 | 0.0066 | 89.1060 | 0.2162 | 19.03 | 0.1984 |
| 120 | Variable | 120.0278 | 0.0135 | 96.4200 | 0.4349 | 19.67 | 0.3644 |

**Supplementary Table S6:** Adjusted means (using emmean package in R studio) for mortality levels in *Dreissena polymorpha* for all nutrient enrichment (Nutrient, in initial grams of osmocotes) and salinity treatments (Salinity, stable of variable). Details are provided for the adjusted means (Adjusted Mean), standard error (SE), degrees of freedom (df), and lower and upper confidence interval on the adjusted mean (respectively Lower CL and Upper CL).

| **Nutrient** | **Salinity** | **Adjusted Mean** | **SE** | **df** | **CI(5%).l** | **CI(5%).u** |
| --- | --- | --- | --- | --- | --- | --- |
| 0 | Stable | 4.05 | 3.78 | 5 | -0.839 | 8.939 |
| 10 | Stable | 4.05 | 11.51 | 5 | -1.177 | 9.277 |
| 20 | Stable | 29.98 | 13.34 | 5 | 6.029 | 53.931 |
| 30 | Stable | 50.36 | 4.38 | 5 | 40.582 | 60.138 |
| 40 | Stable | 60.83 | 12.07 | 5 | 38.426 | 83.234 |
| 50 | Stable | 56.14 | 11.22 | 5 | 29.235 | 83.045 |
| 60 | Stable | 52.65 | 14.91 | 5 | 21.454 | 83.846 |
| 70 | Stable | 96.51 | 6.78 | 5 | 93.243 | 99.777 |
| 80 | Stable | 79.93 | 9.55 | 5 | 66.605 | 93.255 |
| 90 | Stable | 81.70 | 10.08 | 5 | 70.384 | 93.016 |
| 100 | Stable | 84.06 | 4.19 | 5 | 80.288 | 87.832 |
| 110 | Stable | 92.82 | 4.16 | 5 | 86.157 | 99.483 |
| 120 | Stable | 80.16 | 5.71 | 5 | 70.921 | 89.399 |
| 0 | Variable | 8.32 | 4.08 | 5 | -4.047 | 20.687 |
| 10 | Variable | 42.12 | 11.51 | 5 | 8.910 | 75.330 |
| 20 | Variable | 62.99 | 13.35 | 5 | 33.656 | 92.324 |
| 30 | Variable | 94.18 | 4.66 | 5 | 85.959 | 102.401 |
| 40 | Variable | 53.63 | 11.99 | 5 | 28.293 | 78.967 |
| 50 | Variable | 96.39 | 12.55 | 5 | 90.135 | 102.645 |
| 60 | Variable | 58.96 | 14.92 | 5 | 28.317 | 89.603 |
| 70 | Variable | 64.79 | 6.06 | 5 | 48.057 | 81.523 |
| 80 | Variable | 62.07 | 9.56 | 5 | 38.190 | 85.590 |
| 90 | Variable | 57.07 | 9.07 | 5 | 33.478 | 80.662 |
| 100 | Variable | 77.76 | 4.18 | 5 | 74.493 | 81.027 |
| 110 | Variable | 80.58 | 4.01 | 5 | 71.913 | 89.247 |
| 120 | Variable | 81.70 | 5.30 | 5 | 71.922 | 91.478 |

**Supplementary Table S7**: Raw means for mortality levels in *Dreissena polymorpha* for all nutrient enrichment (Nutrient, in initial grams of osmocotes) and salinity treatments (Salinity, stable of variable). Details are provided for the means (Mean), and variance.

| **Nutrient** | **Salinity** | **Mean** | **Variance** |
| --- | --- | --- | --- |
| 0 | Stable | 5.33 | 31.11 |
| 10 | Stable | 4.00 | 35.56 |
| 20 | Stable | 30.67 | 746.67 |
| 30 | Stable | 50.67 | 124.44 |
| 40 | Stable | 61.33 | 653.33 |
| 50 | Stable | 54.67 | 942.22 |
| 60 | Stable | 53.33 | 1266.67 |
| 70 | Stable | 95.00 | 11.11 |
| 80 | Stable | 78.67 | 231.11 |
| 90 | Stable | 83.33 | 133.33 |
| 100 | Stable | 83.33 | 14.81 |
| 110 | Stable | 90.67 | 57.58 |
| 120 | Stable | 80.00 | 88.89 |
| 0 | Variable | 8.33 | 159.26 |
| 10 | Variable | 42.67 | 1435.56 |
| 20 | Variable | 61.33 | 1120.00 |
| 30 | Variable | 91.67 | 70.37 |
| 40 | Variable | 56.67 | 1111.11 |
| 50 | Variable | 95.00 | 40.74 |
| 60 | Variable | 60.00 | 1222.22 |
| 70 | Variable | 65.33 | 364.44 |
| 80 | Variable | 61.33 | 742.22 |
| 90 | Variable | 57.33 | 724.44 |
| 100 | Variable | 81.67 | 11.11 |
| 110 | Variable | 81.33 | 97.78 |
| 120 | Variable | 84.00 | 124.44 |


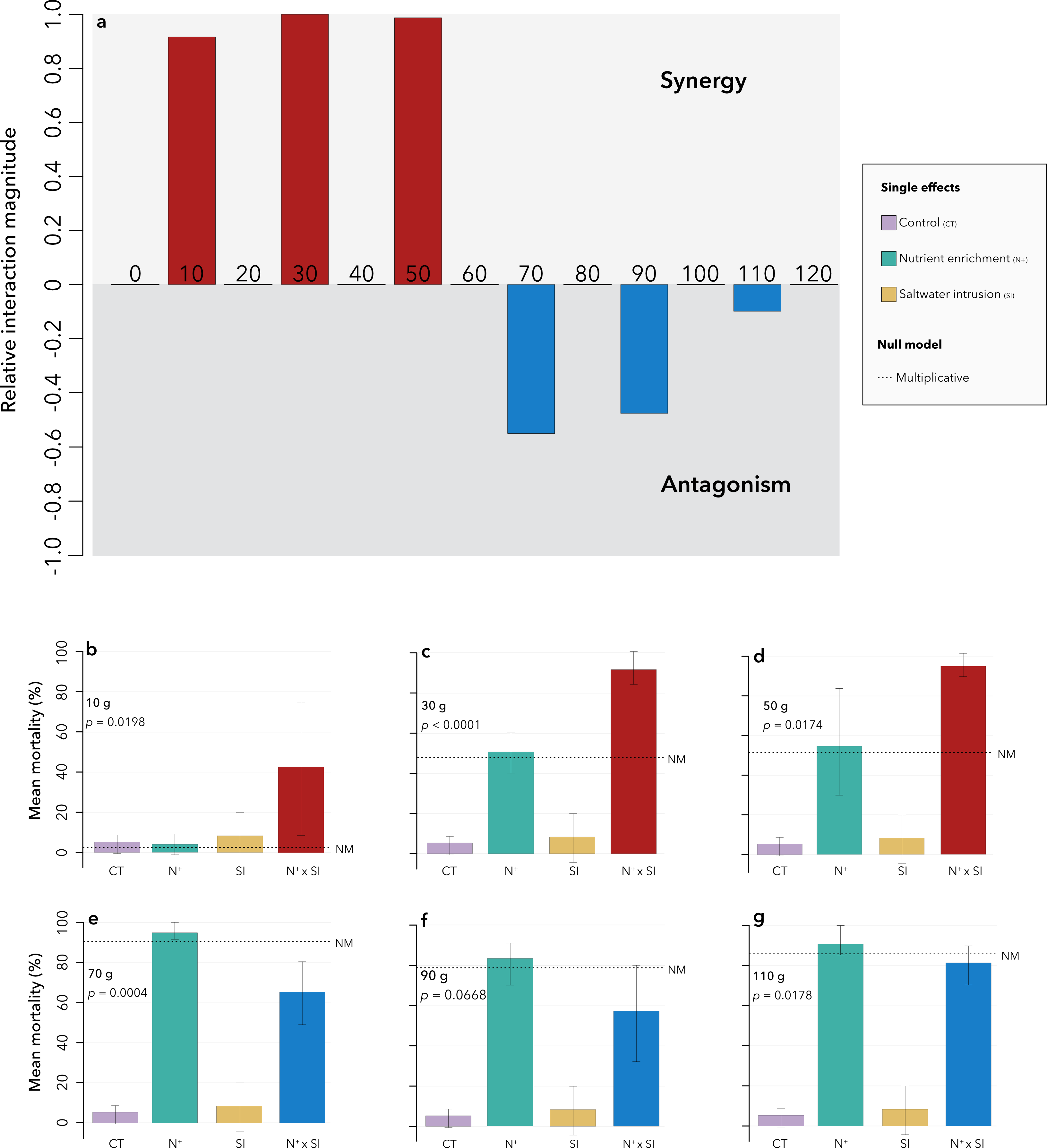


**Supplementary Figure S1:** Stressor interactions (*p* < 0.05) along a nutrient enrichment gradient for mean mortality in the zebra mussel *Dreissena polymorpha* after two months of exposure. (a) Relative interaction magnitude (standardized to 1) between salinity variation and nutrient enrichment. The magnitude was characterized by comparing the combined response of both stressors to the null model. Red bars (upper panel) correspond to synergies and blue bars (lower panel) to antagonistic interactions. In the absence of bars, stressors did not interact. (b to g) mean mortality associated with the different treatments along the nutrient enrichment gradient: control (CT), nutrient enrichment at a given concentration (N^+^), saltwater intrusion (SI) and combined effect of nutrient enrichment and saltwater intrusion (N^+^ x SI). The dashed lines correspond to the level at which a multiplicative null model (NM) would be expected. Error bars correspond to the standard error. Results are shown for the nutrient concentrations where interactions occurred between the two stressors. When stressors did not interact, we deemed a dominance or additive response occurred.
